# Ultra-broadband and high extinction ratio polarization splitter based on triple-tapered directional couplers

**DOI:** 10.1038/s41598-024-67578-6

**Published:** 2024-07-18

**Authors:** Hamed Faramarzi, Afsoun Soltani, Zaker Hossein Firouzeh, Abolghasem Zeidaabadi Nezhad, Reza Safian

**Affiliations:** 1 Department of Electrical and Computer Engineering, Isfahan University of Technology, Isfahan, 8415683111 Iran; 2 IMEC USA, 194 Neocity Way, Kissimmee, FL 34744 USA

**Keywords:** Optics and photonics, Electrical and electronic engineering

## Abstract

The design and numerically investigation of a silicon polarization splitter (PS) is proposed using triple-tapered directional couplers (DCs).The proposed device consists of a triple-tapered DC, a triple-bent DC at through port, and a tapered waveguide at cross port. The coupling length of the device is 20.5 µm. At the 1550 nm wavelength, the realized PS exhibits the extinction ratio (ER) values of 35.7 dB and 37.35 dB as well as the insertion loss values of < 0.58 dB and < 0.4 dB for the TE and TM polarization modes, respectively. Furthermore, the device’s bandwidth for ER > 20 dB and ER > 25 dB are 170 nm and 136 nm, respectively.

## Introduction

Photonic integrated circuits (PICs), thanks to their compact footprint and low power consumption, are widely used in optical communication applications^1^. Silicon on insulator (SOI) is a common platform used in PIC technology, which has drawn considerable interest through the high refractive index contrast between silicon and silicon dioxide (SiO$_{2}$)^2^. This feature facilitates the development of highly compact devices that can be integrated onto a single chip. However, despite the advantages of high index contrast in silicon waveguides, they suffer from a large modal birefringence, which means that integrated devices made from them can be very sensitive to the polarization of the light^2,3^. To overcome the challenge of polarization dependence, some polarization handling devices such as polarizers^4^, polarization splitters (PSs)^5^, polarization rotators^6,7^, and polarization splitter–rotators^8^ have been introduced.

Considered a crucial element for separating or combining TE and TM light, the PS plays an important role in PICs^9^. In broadband telecommunication systems, PSs with high bandwidth, high extinction ratio (ER) and low loss are required^10^. Up to now, different PS designing approaches have been proposed including Y-branch^11^, Mach–Zender interfrometer (MZI)^12–14^, multimode interferometer (MMI)^15,16^, photonic crystal (PhC)^17^, and directional coupler (DC)^18–20^. Large footprint in PSs based on MMI and MZI, large propagation loss as well as the complexity of fabrication process in PhC-based PSs^21^, and the limited bandwidth in PSs based on DC are important drawbacks of each approach^22^. However, DC-based PSs with their compact footprint, fabrication tolerant, and straightforward design procedure are more interesting^23^.

So far, a multitude of DC configurations have been investigated for the design of DC-based PSs, encompassing regular asymmetric^24^, bent^25^, sub-wavelength grating (SWG)^26^, slot waveguide^27^, adiabatic^28^, and triple waveguide^29,30^ approaches. It should be noted that, each of these configurations has certain limitations that need to be carefully considered during the design process. For instance, bent DCs pose a challenge in achieving an ER greater than 20 dB^31,32^. Furthermore, regular and adiabatic DC forms suffer from dimensional fluctuations during the fabrication process^21^, and significant footprint^33^, respectively. In addition, SWG and slot waveguide DCs need complicated fabrication requirements compared to the other types^10,34^. In the case of triple waveguide DCs, the increased size and design complexity are the challenging points. However, precise phase control, potentially wider bandwidths, and preparing more degree of freedom^35–37^ lead to select them as an appropriate offer for the propose of this study. Up to now, several DCs based on triple waveguide PSs with different performance characteristics have been reported. For instance, a conventional DC-based PS has been proposed to reach ER > 20 dB along with a bandwidth of 60 nm^25^. In addition, a triple waveguide DC has been reported with an impressive ER of over 40 dB at 1550 nm as well as a bandwidth of 70 nm for an ER > 20 dB^36^. Furthermore, a triple-bent-waveguide DC has been used to design and fabrication of a broadband silicon PS with a 90 nm bandwidth meeting the ER > 20 dB^37^.

In this paper, the design and numerically investigation of a broadband silicon PS (170 nm) with a high ER (> 35 dB) are proposed. The proposed device consists of a triple-tapered DC, a triple-bent DC at through port, and a tapered waveguide at cross port. The coupling length ($L_{c}$) is obtained as compact as 20.5 µm. In addition, the minimum waveguides separation and the minimum bend radius are 250 nm and 20 µm, respectively, facilitate the fabrication process. The simulation analyses performed at wavelength 1550 nm for TE and TM polarization modes result the ER values of 35.7 dB and 37.35 dB, respectively. Additionally, the evaluated insertion loss (IL) values at this wavelength are 0.58 dB (for TE) and 0.4 dB (for TM). Moreover, the attained bandwidth for ER > 20 dB and ER > 25 dB are 170 nm and 136 nm, respectively.

## Design and simulation results

Based on the theory of coupling mode, for two adjacent waveguides that form a DC, there are two even (symmetric) and odd (asymmetric) modes with the corresponding effective index $n_e$ and $n_o$, respectively. The coupling process from a waveguide to the other one take place at the coupling length $L_{c}$ expressed as^38^:

1 $$\begin{aligned} L_c=\frac{\lambda }{2(n_{e}-n_{o})} \end{aligned}$$

where $\lambda$ is the working wavelength. Since the effective index of TE and TM polarization modes are different in SOI waveguides, the calculated $L_{c}$ for these modes is not the same. This difference in coupling lengths allows SOI-based DCs to separate light based on its polarization, making them work like polarization splitters. Studies have shown that adding an extra waveguide to the standard DC design, creating a three-waveguide DC, can improve coupling efficiency^39,40^. In this case, three distinct single-mode waveguides lead to three different propagation modes for both TE and TM polarizations, consisting of two symmetric modes with propagation constants $\beta _A$ and $\beta _B$ as well as an asymmetric mode with propagation constant $\beta _C$^41^. The coupling length for these waveguides represented as^40^:

2 $$\begin{aligned} { L_c=\frac{\lambda }{n_{A}-n_{B}} =\frac{\lambda }{2(n_{A}-n_{C})}} \end{aligned}$$

where $n_A$ and $n_B$ are the effective indices for the symmetric modes and $n_C$ is the asymmetric mode’s effective index. For the propose of using a triple-waveguide DC as a PS, it is necessary to satisfy the phase-matching condition $(2\beta _C=\beta _A+\beta _B)$ for one of the polarization modes (TE/TM), which leads to the complete coupling of this polarization into the DC’s cross port. It should be noted that, the difference in the coupling length of TE and TM polarizations does not allow the other polarization to experience coupling process and thus moves along the DC’s through port. In this work, the design procedure is performed to meet the phase-matching condition for TM polarization.

**Figure 1:**
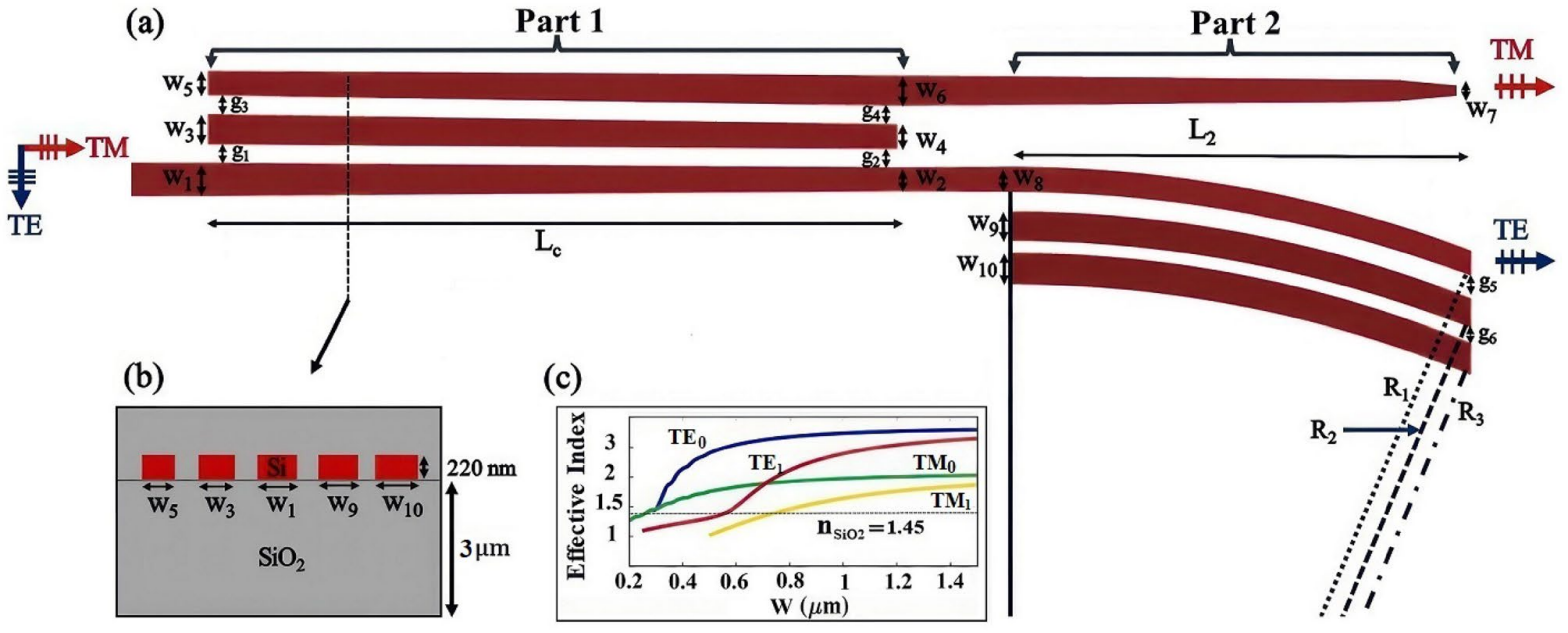
(**a**) 2D schematic diagram of the proposed PS: Part 1 (a triple-tapered DC ) and Part 2 (a triple-bent DC at through port and a tapered waveguide at cross port). (**b**) Cross-sectional view of the proposed PS. (**c**) Effective index graphs for a buried silicon nanowire waveguide in SiO$_{2}$.

Figure 1a illustrates 2D schematic diagram of the proposed PS, which comprises two main parts. The first part includes a triple-tapered DC (DC1) consisting of three linearly tapered waveguides. The second part consists of a triple bend DC (DC2) embedded at through port to effectively eliminate any residual TM polarization, and a tapered waveguide at cross port to mitigate the undesired TE polarization.

In all the simulations, wavelength $\lambda$ = 1550 nm and the buried 220 nm silicon nanowire waveguides ($\hbox {n}_{Si}$ = 3.45) into the SiO$_{2}$ ($\hbox {n}_{{\mathrm{SiO}}_{2}}$ = 1.45) are assumed. The thickness of silicon oxide for the upper and lower cladding layers are 1.5 µm and 3 µm, respectively. The cross-sectional view of the proposed device is shown in Fig. 1b. The first step in designing the waveguides is to identify the width enabling single-mode light propagation. To this purpose, the effective index graphs of a buried silicon nanowire waveguide in $\hbox {SiO}_{2}$ are illustrated in Fig. 1c. The width w $\simeq$ 600 nm is the maximum width that the higher order modes $\hbox {TE}_{1}$ and $\hbox {TM}_{1}$ can not be propagated in the waveguide. It is due to their effective index values, which are approximately near and less than the cladding index ($\hbox {n}_{{\mathrm{SiO}}_{2}}$ = 1.45), respectively. Therefore, the maximum width of 600 nm is considered for the design of all waveguides in this work.

In the next step, three dimensional finite-difference time-domain (3D FDTD) simulation is used to evaluate the geometrical parameters. It should be noted that, the optimized width and gap parameters in asymmetric DCs with two waveguides are usually estimated by the effective index method (EIM)^18,42^. However, due to the complex distribution of the refractive index, EIM is not compatible with arbitrarily shaped waveguides^43^, like the ones proposed in this work. In order to calculate the input and bridge waveguides’ widths, the waveguide’s separation distance is initially assumed to be 370 nm, which results in $\hbox {w}_{1}$ = 560 nm, $\hbox {w}_{2}$ = 400 nm, $\hbox {w}_{3}$ = 500 nm, and $\hbox {w}_{4}$ = 400 nm. In addition, the widths of the cross port tapered waveguide ($\hbox {w}_{5}$ and $\hbox {w}_{6}$) are optimized via a parameter sweep to achieve maximum efficiency for the TM polarization mode. The optimal values are found to be $\hbox {w}_{5}$ = 485 nm, $\hbox {w}_{6}$ = 530 nm, and $\hbox {w}_{7}$ = 485 nm. Moreover, further optimization is performed for the distance between the waveguides, which gives $\hbox {g}_{1}$ = 355 nm, $\hbox {g}_{2}$ = 400 nm, $\hbox {g}_{3}$ = 350 nm, and $\hbox {g}_{4}$ = 360 nm. Using these optimized parameter values, the calculated coupling length for TM polarization mode is $L_{c}$ = 20.5 µm. Since the coupling length for TE mode is longer than TM mode, the substantial restriction of TE mode in the through port as well as the strong coupling for TM mode are guaranteed. It should be noted that the operating wavelength range for this device varies between 1430 and 1670 nm, which can supports E-, S-, C-, L-, and U- frequency bands.

To characterize the properties of the proposed PS the transmission power, ER, and IL for both TE and TM polarization modes are evaluated using the following equations^36^:

3 $$\begin{aligned} T_{through}&= \frac{1}{4} \left[ 1+cos^2\frac{\left( \beta _A-\beta _B\right) }{2}z-cos\left( \beta _A-\beta _C\right) z-cos\left( \beta _C-\beta _B\right) z\right] \end{aligned}$$

4 $$\begin{aligned} T_{cross}&= \frac{1}{4} \left[ 1+cos^2\frac{\left( \beta _A-\beta _B\right) }{2}z+cos\left( \beta _A-\beta _C\right) z+cos\left( \beta _C-\beta _B\right) z\right] \end{aligned}$$

5 $$\begin{aligned} ER_{through \, (cross)}&= 10log_{10}\left[ T_{through \, (cross)}/T_{cross \, (through)}\right] \end{aligned}$$

6 $$\begin{aligned} IL_{through \, (cross)}&= -10log_{10}\left[ T_{through \,(cross)}\right] \end{aligned}$$

**Figure 2:**
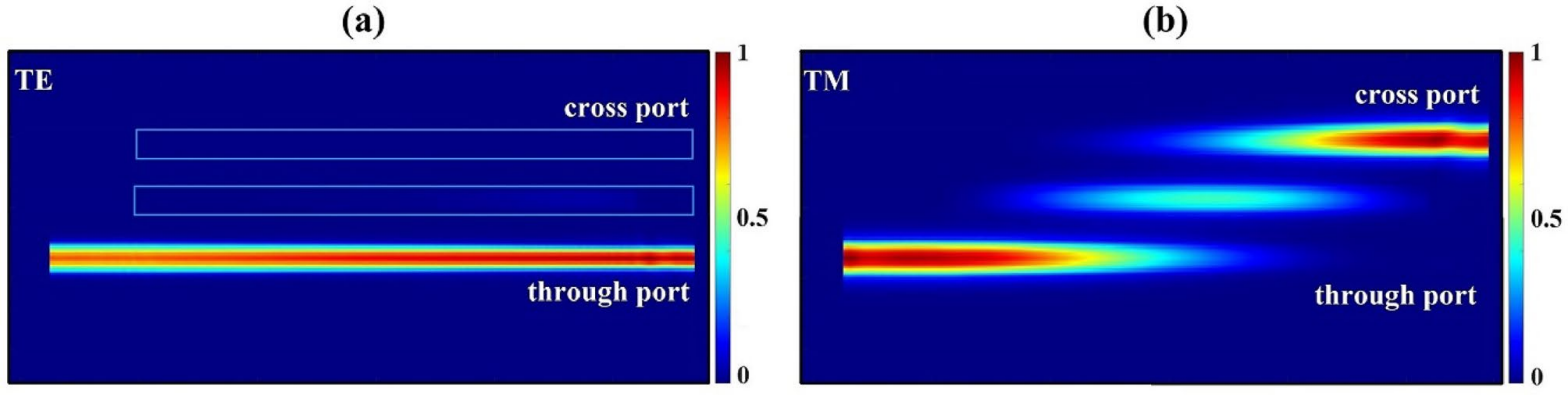
Beam propagation profiles for the TE and TM polarization modes in the designed triple-tapered DC at 1550 nm.

Figure 2a and b illustrate the simulated beam propagation profiles for the TE and TM polarization modes in the designed triple-tapered DC at 1550 nm, respectively. From the profiles, the input TE light goes directly into the through port without coupling, while the input TM light gradually couples into the cross port.

**Figure 3:**
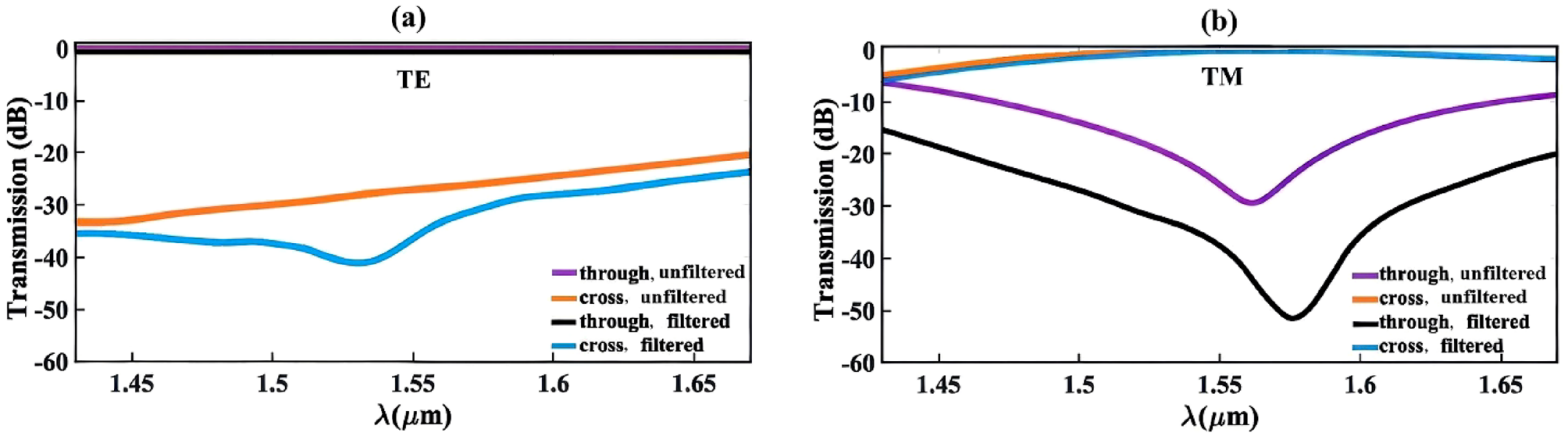
The calculated transmission in output ports for (**a**) TE and (**b**) TM polarization modes.

As it can be seen in Fig. 1a, the designed PS includes a triple-bent DC and a tapered waveguide at its through and cross ports, respectively. These structures are employed to filter the undesired TM and TE modes in their corresponding ports, which leads to improve the bandwidth of the PS. Figure 3a and b show the calculated transmission for the input propagated TE and TM polarization beams with the wavelength in range 1430–1670 nm for both with and without filtering structures, respectively. Due to the intrinsic wavelength dependence of the evanescent coupling length, the TM mode transmission is wavelength-sensitive, which is clearly inferred from Fig. 3b. However, in unfiltered structure the designed PS has a bandwidth as broad as 195 nm (1475–1670 nm) to achieve a transmission efficiency of < 2 dB. As it can be seen in Fig. 3a, the transmission efficiency is less wavelength-sensitive for TE mode, so that the efficiency < 0.28 dB is attainable over a broad wavelength range from 1430 to 1670 nm. The evaluated transmission at wavelength 1550 is also less than 0.22 dB for both polarizations. It is clear from the figures that the utilization of the filters improves the device’s efficiency for both TE and TM polarizations at cross and through ports, respectively.

**Figure 4:**
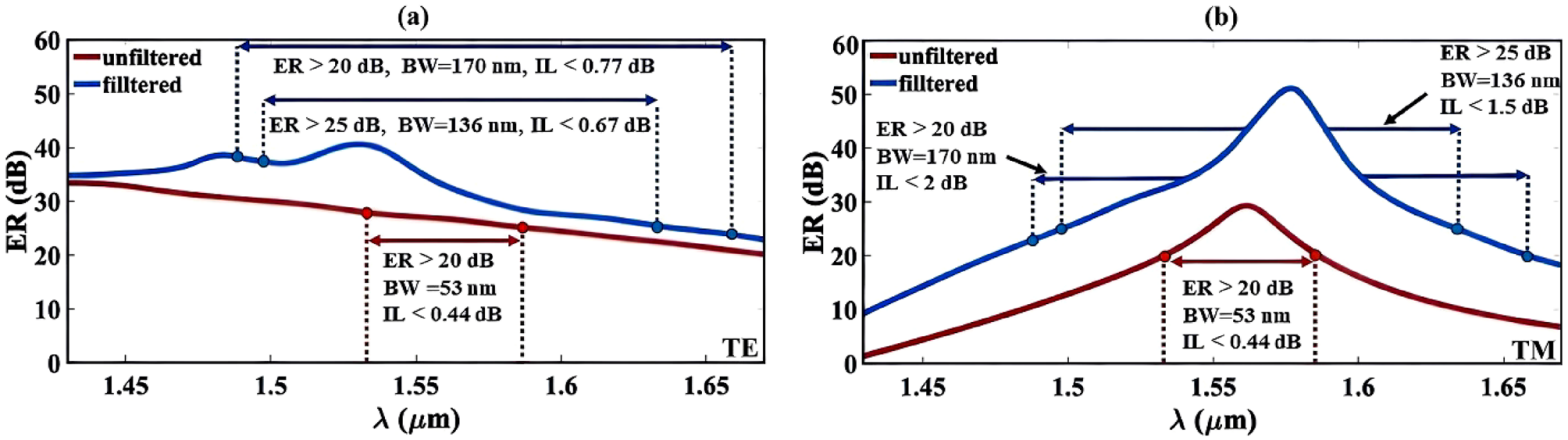
The calculated ER in output ports for (**a**) TE and (**b**) TM polarization modes. The use of filtering structures at the output ports leads to improve the PS’s bandwidth.

The impact of using the filters on the calculated ER is depicted in Fig. 4a and b, for the TE and TM polarization modes, respectively. In the absence of filters, the device demonstrates the ER values of 27 dB and 25 dB at wavelength 1550 nm for the TE and TM polarizations, respectively. In addition, IL is lower than 0.22 dB at this wavelength for both polarizations. Furthermore, the device’s bandwidth to achieve ER > 20 dB and IL < 0.44 dB is 53 nm in this case. Applying the filters in both through and cross ports lead to attain a bandwidth as broad as 170 nm (1488–1658 nm) for ER > 20 dB and IL values less than 0.77 dB and 2 dB for TE and TM modes, respectively. In addition, for ER > 25 dB a bandwidth 136 nm (1497–1633 nm) is achieved with IL < 0.67 dB for TE mode and IL < 1.5 dB for TM mode. Moreover, at the wavelength 1550 nm, the TE polarization exhibits ER = 35.7 dB, and TM polarization reaches a remarkable ER value of 37.35 dB with IL < 0.58 dB.

**Figure 5:**
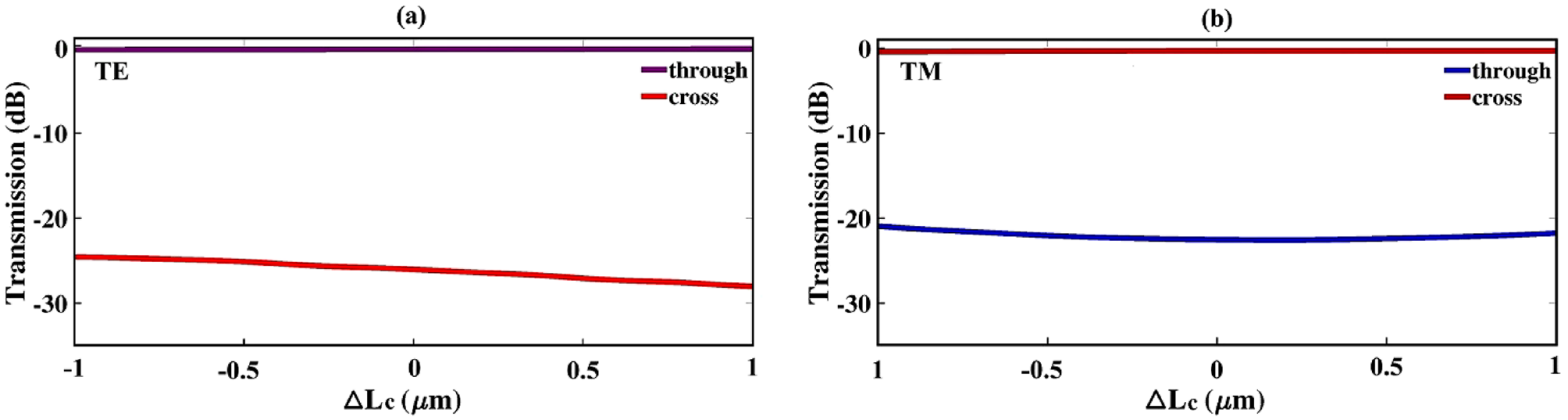
Transmission sensitivity to the variation of $L_{c}$ for both through and cross ports: (**a**) TE mode, and (**b**) TM mode.

**Figure 6:**
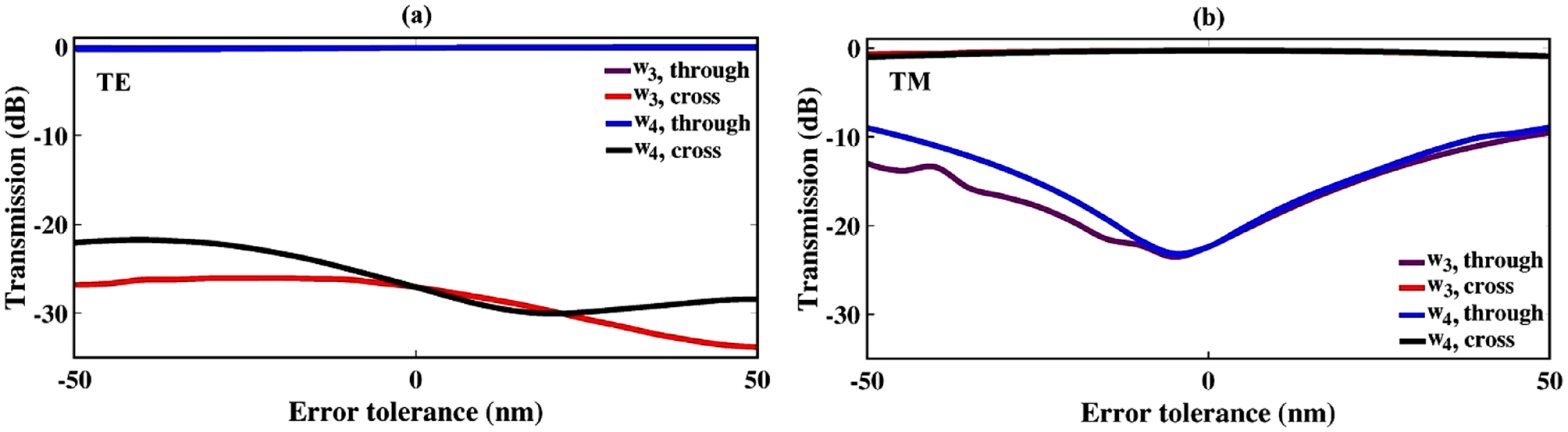
Fabrication error tolerance; when a deviation of ± 50 nm is applied into the bridge’s widths of $\hbox {w}_{3}$ and $\hbox {w}_{4}$ for (**a**) TE and (**b**) TM modes.

**Figure 7:**
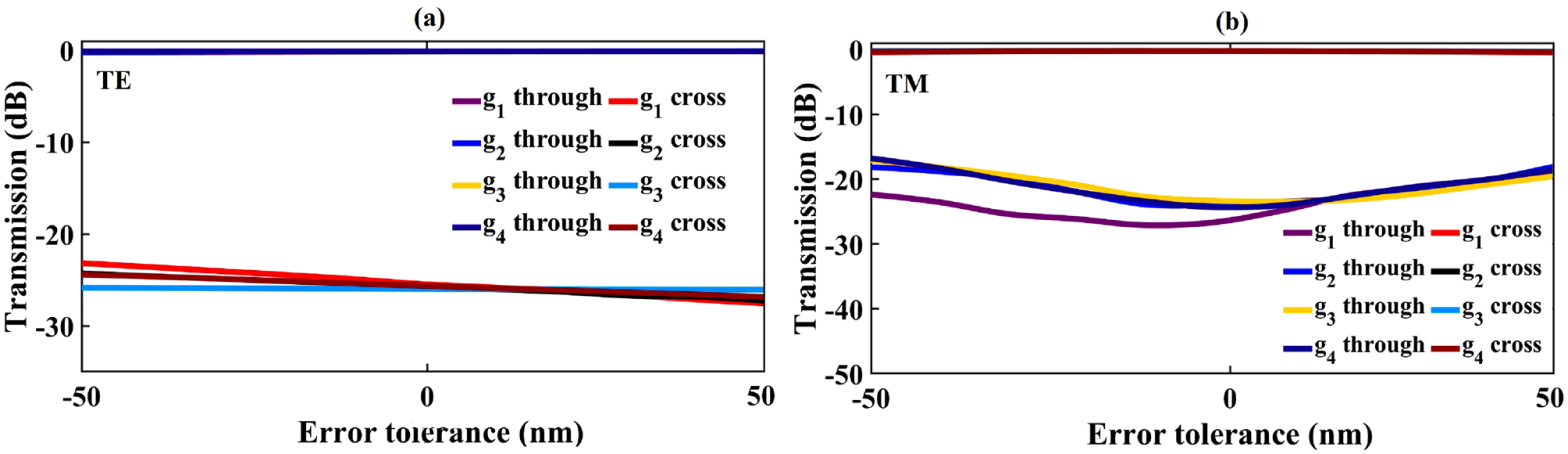
Fabrication error tolerance; when a deviation of ± 50 nm is applied into the coupling gap parameters ($g_1$ to $g_4$) for (**a**) TE and (**b**) TM modes.

For the purpose of seeking the effect of the coupling length’s variation on transmission efficiency, a deviation of $\Delta Lc$ =  ± 1 µm is applied to the proposed structure. The results are depicted in Fig. 5. As shown in this figure, despite there are slightly changes in transmission values for TE in cross and TM in through ports, other graphs remain essentially flat over the deviation range. Accordingly, the effect of coupling length’s variation into the transmission efficiency assumed to be negligible. In order to investigate the fabrication error tolerance, the bridge’s widths ($\hbox {w}_{3}$ and $\hbox {w}_{4}$) are also simultaneously varied within $\Delta$ $\hbox {w}_{3}$ = $\pm 50$ nm and $\Delta$ $\hbox {w}_{4}$ = $\pm 50$ nm, with fixing the waveguides’ widths and gaps. As illustrated in Fig. 6, the efficiency of the TM mode is more sensitive to the changes in the bridge’s widths than the TE mode. The study of the fabrication error tolerance is also performed for the coupling gap parameters ($g_1$ to $g_4$). The results are shown in Fig. 7. This figure describes a relatively large tolerance for both TE and TM polarization modes.

In the end, a comparative analysis among the various reported PSs is presented in Table 1. It can be seen that the proposed ultra-broadband PS outperforms other similar triple waveguide DC-based PSs, achieving high ER values, exceeding 20 dB and 25 dB across the entire bandwidths of 170 nm and 136 nm, respectively. Moreover, our proposed device shows IL no more than 0.58 dB and 0.4 dB for TE and TM polarization modes, respectively, at the working wavelength.

**Table 1:** Comparison of different types of PSs with the proposed device.

References	Structures	ER at 1550 nm (dB)		IL at 1550 nm (dB)		$BW_{20\,{\mathrm{dB}}}$ (nm)	$BW_{25\,{\mathrm{dB}}}$ (nm)	$L_{c}$ (µm)
		TM	TE	TM	TE			
^28^	Three-waveguide DC*	40.79	39.01	0.35	0.35	70	40	29.4
^29^	Cascade triple-bent DC	42	42	< 1	< 1	90	70	13
^35^	Triple WG short bridge	22.5	22.9	2.1	1.8	NA	NA	7.5
^20^	Bent DC	35	35	0.35	0.35	135	110	20
^34^	Triple WG (SWG bridge)	26.38	28.48	0.27	0.15	60	NA	6.5
This work	Triple-tapered DC	37.35	35.7	0.4	0.58	170	136	20.5

*NA* not available.

*Measurement result. $BW_{20\,{\mathrm{dB}}}$ (nm), $BW_{25\,{\mathrm{dB}}}$ (nm) are the bandwidths for ER > 20 dB and ER > 25 dB.

## Conclusion

An ultra-broadband and high ER (> 35 dB) PS, based on triple-tapered DCs, is designed and numerically investigated. The proposed structure consists of a triple-tapered DC, a tapered waveguide at cross port, and a triple-bent DC at through port, which gives a high degree of freedom for optimization. The waveguide widths for the proposed device are chosen appropriately to satisfy the phase-matching condition for TM polarization. The coupling length is 20.5 µm. The calculated ER at the central wavelength 1550 nm is 37.35 dB for TM and exceeds 35.7 dB for TE polarization mode. Furthermore, the attained bandwidth is as broad as 170 nm for ER > 20 dB with IL values less than 0.77 dB and 2 dB for TE and TM modes, respectively. In addition, for ER > 25 dB a bandwidth 136 nm is achieved with IL < 0.67 dB for TE mode and IL < 1.5 dB for TM mode. The proposed ultra-broadband PS has great potential applications in PICs. The device is CMOS-compatible and it can be easily realized by the mature fabrication technology.

## Data Availability

Data supporting this study are available from the corresponding author on reasonable request.
